# Patient specific modeling of palpation‐based prostate cancer diagnosis: effects of pelvic cavity anatomy and intrabladder pressure

**DOI:** 10.1002/cnm.2734

**Published:** 2015-08-19

**Authors:** Javier Palacio‐Torralba, Elizabeth Jiménez Aguilar, Daniel W. Good, Steven Hammer, S. Alan McNeill, Grant D. Stewart, Robert L. Reuben, Yuhang Chen

**Affiliations:** ^1^ Institute of Mechanical, Process and Energy Engineering, School of Engineering and Physical SciencesHeriot‐Watt UniversityEdinburghEH14 4ASUK; ^2^ Servicio de Oncología MédicaHospital 12 de OctubreMadrid28041Spain; ^3^ Edinburgh Urological Cancer Group, Division of Pathology Laboratories, Institute of Genetics and Molecular MedicineUniversity of Edinburgh, Western General HospitalCrewe Road SouthEdinburghEH4 2XUUK; ^4^ Department of Urology, NHS LothianWestern General HospitalCrewe Road SouthEdinburghEH4 2XUUK

**Keywords:** patient‐specific modeling, tissue diagnostics, palpation, soft tissue, prostate cancer, cancer diagnosis

## Abstract

Computational modeling has become a successful tool for scientific advances including understanding the behavior of biological and biomedical systems as well as improving clinical practice. In most cases, only general models are used without taking into account patient‐specific features. However, patient specificity has proven to be crucial in guiding clinical practice because of disastrous consequences that can arise should the model be inaccurate. This paper proposes a framework for the computational modeling applied to the example of the male pelvic cavity for the purpose of prostate cancer diagnostics using palpation. The effects of patient specific structural features on palpation response are studied in three selected patients with very different pathophysiological conditions whose pelvic cavities are reconstructed from MRI scans. In particular, the role of intrabladder pressure in the outcome of digital rectal examination is investigated with the objective of providing guidelines to practitioners to enhance the effectiveness of diagnosis. Furthermore, the presence of the pelvic bone in the model is assessed to determine the pathophysiological conditions in which it has to be modeled. The conclusions and suggestions of this work have potential use not only in clinical practice and also for biomechanical modeling where structural patient‐specificity needs to be considered. © 2015 The Authors. International Journal for Numerical Methods in Biomedical Engineering published by John Wiley & Sons Ltd.

## 1 Introduction

Cross‐disciplinary research between engineering and medicine has resulted in significant advances in clinical diagnostics and treatments for cancer such as magnetic resonance imaging (MRI) and radiotherapy. More importantly for this work, computational modeling has become a useful predictive tool, which has been proven to benefit clinical practice in a number of ways, including surgery planning 1, clinician training 2 and preventive medicine 3. Other biomedical and biological areas, such as tissue engineering 4, biomaterials research 5 and cell mechanics 6, have also been advanced using *in‐silico* modeling. However, the vast majority of the studies reported in the literature use general models, often with insufficient patient‐specific input, in such scenarios as arterial clamping 7, prostate cancer diagnostics 8 or cornea pinching 9. Nowadays, it is widely accepted that patient specificity plays a vital role in the effectiveness of predictive models 10, 11, 12. Therefore, it is of great importance to examine the sensitivity of any model to patient‐specific parameters, especially when quantitative information is required to make clinical decisions.

There are some examples in the literature of how patient specific modeling has successfully enhanced clinical diagnostics and treatment. Bone tissue has received significant interest in the past decade because of the social and economic impact of diseases including osteoporosis and osteoarthritis and the correction of traumas, such as hip fracture. Garijo et al. 13 used artificial neural networks, support vector machines and linear regression to predict loads in bone when taking into account patient specific features such as proximal femur geometry. Kerner et al. 14 investigated whether bone loss following total hip arthroplasty could be explained by strain remodeling using patient specific geometries and bone densities. For osteoporosis, Schileo et al. 15 proposed a framework which included patient specific geometries and material properties to determine an index based on tissue density and Young's modulus to predict the strain in bones and, consequently, the probability of fracture.

Other examples of patient specific modeling include planning of clinical treatment such as radiotherapy and cryosurgery. The side effects of radiotherapy, for example, can be mitigated by predicting the movement of the healthy organs that surround the malignant tissue. In this respect, Scaife et al. 16 have developed a framework, which takes patient uniqueness into account, to determine the position and absorbed dose of the rectum during radiation treatment to avoid unnecessarily extreme exposures. In the area of cryosurgery, which uses probes to cool down tissues below a certain threshold (e.g. for prostate cancer surgery), Zhang et al. 17 have modeled the temperature distribution taking into account patient‐specific differences in thermal properties as well as the heating from the urethral warming catheter, which is inserted to reduce the risk of damage to the tissue of the urethra. This approach allows a safer and more reliable procedure where the malignant tissue can be eliminated while maintaining the integrity of surrounding healthy tissue and adjacent structures.

Patient specific modeling has also been quite successful in other clinical areas in recent years 18, 19. Gasser and co‐workers proposed a systematic methodology to estimate the risk of rupture of abdominal aorta aneurysms which has been employed in clinical practice 20. In this framework, the geometry of the blood vessels was obtained from the patient using CT scans, although average values were used for the mechanical properties. Patient specific models have greatly assisted in understanding the mechanisms behind the breakdown of aortic aneurisms 21 and consequently the prediction of rupture risk 22 thus making a significant contribution in preventive cardiovascular medicine.

Prostate cancer is one of the most widespread cancers in men 23. It has been shown that the mechanical properties of prostatic tissue vary in the presence of pathological conditions including benign prostate hyperplasia and prostate cancer. The most common techniques to diagnose prostate cancer include MRI, elastography and digital rectal examination. In addition to the detection of anomalies in the prostate, MRI allows the study of other surrounding organs such as the seminal vesicles 24. Elastography has also been used widely to help diagnose prostate cancer 25, 26, 27 and is based on the detection of relative displacements of stiffer and less stiff parts of the organ. Digital rectal examination (DRE) is a procedure where the practitioner palpates the patient's prostate from the rectal wall, looking for lumps and changes in roughness as the indication of presence of cancer. However, this technique remains rather qualitative and is subject to inter‐clinician variations. To improve this situation instrumented digital rectal examination (IDRE) systems have been proposed and developed 28, 29. As opposed to DRE, where the practitioner only receives qualitative information through the finger, in IDRE the force feedback of palpation is measured and recorded for an enhanced analysis as well as being objective and recordable. This has led to the development of methodologies to identify areas suspected of being malignant and to offer the possibility of distinguishing diffuse and/or indolent tumors 30. It is therefore of great clinical importance to incorporate patient‐specific data into *in‐silico* instrumented DRE, which could significantly improve its effectiveness in quantitative prediction by investigating sensitivity to patient specific parameters and so guide clinical practice in the interpretation of IDRE.

The aim of this paper is twofold: first to present a computational framework for modeling the male pelvic cavity with a focus on prostatic diagnostics using palpation, and, secondly, to provide a ‘worked example’ of quantitative analysis of patient‐specificity including inter‐patient structural differences, intrabladder pressure and the influence of the pelvic bone, all in order to improve the efficacy of IDRE.

## 2 Materials and Methods

### 2.1 Patient selection

**Figure 1:**
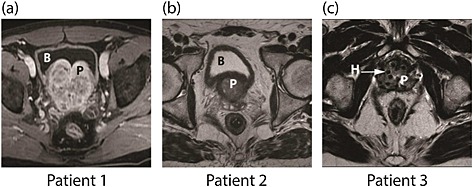
Typical MR images of the three patients selected for the study, showing patient‐specific structural features. B and P indicate the location of the bladder and prostate, respectively. (a) Patient 1: an enlarged prostate compresses the bladder; (b) Patient 2: the inferior of the bladder is tightly wrapped around the superior part of the prostate; (c) Patient 3: prostate contains holes (H) because of brachytherapy treatment.

Three male patients with different anatomical and pathological conditions were selected in this study to investigate how patient‐specificity influences the outcome of the instrumented digital rectal examination for the purpose of prostate cancer diagnosis. Figure 1 shows representative MR images of each patient. From Figure 1(a) it can be seen that Patient 1 has an enlarged prostate which suggests adenomatous hyperplasia. An imprecise marginal area suspected of being malignant was identified at the vertex and in the anterior zone. A distinctive structural feature of this patient is an enlarged prostate that compresses the bladder causing pollakiuria, a condition that results in high frequency of urination. The MRI scan of Patient 2 in Figure 1(b) shows invasion of cancerous tissue into the peripheral and posteriocentral zones, which suggests a neoplastic process prolongated to the periurethral zone. Ganglionar and indeterminate iliac bone lesions can also be observed in the scan, although they are not modeled in this study. The clinical history of Patient 3 indicates that brachytherapy was conducted, where cancerous tissue was destroyed by the effect of radioactive seeds inserted into the tissue. The MRI scan shown in Figure 1(c) indicates loss of central–peripheral differentiation of the gland as well as thinning of the posterior right capsular limits, continued with the seminal vesicles and neurovascular plexus. Ganglionar disease and right sacral bone lesions are also present but not modeled. These observations are consistent with recurrent prostate cancer with at least stage T3b (tumor spread to the seminal vesicles), N1 (cancerous cells present in the lymph nodes) and M1b (metastasis to the bone), using the TNM (Tumor size, nearby lymph node involvement, distant metastasis) classification 31. T2‐weighted, 1.5‐Tesla MRI scans, with a resolution of 3.0 mm in the axial plane (all patients) and 0.7813‐mm (Patients 1 and 2) and 0.651 mm (Patient 3) resolution in the sagittal and coronal planes were used to reconstruct the 3D model in ITK‐SNAP 32.

### 2.2 Organ and physiological modeling

The male pelvic cavity contains the end part of the large intestine, the urinary bladder, the prostate, the seminal vesicles and the pelvic bone plus some other, minor structures. Its patient‐specific modeling presents various challenges, depending on the clinical application, including decisions on; which organ(s) to model, how they interact with each other and also the initial conditions of tissue. The models proposed in this paper are aimed at understanding the effects of patient‐specific features in IDRE as well as presenting a diagnostic framework for patient specific modeling of prostatic diseases. Intrabladder pressure (IBP) and the pathological condition of the prostate are key parameters, and their influence on the effectiveness of IDRE will be investigated through predictive modeling in this study. Moreover, the effect of inclusion of the pelvic bone in the *in‐silico* IDRE model will be assessed. It should be noted here that, although there are a few clinical parameters that can be used to optimize the procedure, such as the position of the patient during the examination, the margin of potential clinical usefulness is limited because of patient discomfort. IBP, however, can be easily altered during clinical examinations. This can be controlled by the patient's actions prior to the examination or with the help of a catheter. Table 1 shows the different IBPs that were adopted in this study to model a full and empty bladder 33. Bladders of all three patients were empty prior to the MRI scan, and the reconstructed 3D organs are considered as undeformed and unstressed.

**Table 1:** Variation of intrabladder pressure (IBP) subjected to different volumes of urine. Data were taken from Chiumello et al. 33, where IBP was used as an indicator of intra‐abdominal pressure.

Volume of saline (ml)	IBP (mmHg)	IBP (MPa)
50	9.5	1.267 × 10^−3^
200	27.1	3.613 × 10^−3^

In the rest of this paper, the pressure corresponding to a bladder content of 50‐ml urine will be referred to as low intrabladder pressure (LIBP) and the content of 200 ml as high intrabladder pressure (HIBP). During normal activities, the rectum is subjected to a certain internal pressure 34. However, because of the insertion of the instrumented system (or finger, in the case of digital rectal examination), it will be considered to be at ambient pressure during the procedure.

### 2.3 Material modeling

Like most biological tissues, the fascia, bladder, prostate and rectum exhibit viscoelastic behavior 35, 36, 37, 38. Nevertheless, under certain loading conditions, their mechanical behavior can be considered to be elastic, especially when the strain rate is very low as is the case for DRE. When the strain rate is very low the viscous component can be neglected, and the behavior can be modeled using the apparent stiffness. Subjected to such a quasi‐static loading the observed stiffness is often referred to as long‐term modulus. This assumption, which simplifies the experiments and modeling, has been widely used in the literature 8, 39, 40 and will be used in this study. To ensure numerical stability, the mechanical properties of the tissues are modeled by non‐linear hyperelastic strain energy density functions that mimic the behavior shown in Table 2.

**Table 2:** Mechanical properties and material models used for tissues considered in this study.

Material	Equivalent Young's modulus (kPa)	Hyperelastic model	Reference
Rectum	10	Neo‐Hookean	42
Healthy prostatic tissue	17	Ogden second order	37
Cancerous prostatic tissue	34	Ogden second order	37
Bladder	15	Neo‐Hookean	42
Fascia	15	Neo‐Hookean	42
Bone	~6 × 10^6^	Neo‐Hookean	43

**Figure 2:**
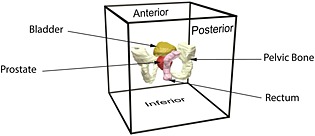
Computational model where organs are embedded in a box of fascia. The displacement of the anterior side of the box is constrained.

The organs under consideration are embedded in a ‘box’ of fascia to allow the modeling to concentrate on studying the effect of patient specific features. The size of this box (300 × 300 × 300 mm) is chosen to be comparable to the size of patient's pelvic cavity, big enough so that the effect from boundary conditions that prevent rigid body motions becomes negligible while keeping the computational cost to a minimum. In this study, it is considered that the patient is bent at the waist over a table during examination. Therefore, in the proposed model, the displacement at the anterior side of the box is constrained. Figure 2 shows the organs of patient 2 embedded in the box and the anterior side where the boundary conditions are applied.

**Figure 3:**
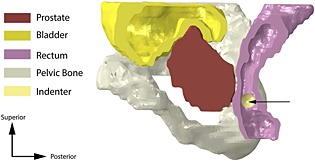
Schematic of the 3D reconstructed model of Patient 2. The arrow indicates the direction of indentation.

The patient‐specific models were simulated in ABAQUS (Dassault Systemes, Vlizy‐Villacoublay, France). The organs were meshed with linear hybrid tetrahedral elements where the hydrostatic pressure is considered as an independent variable and coupled with the displacement using the constitutive model. This approach is needed to model such quasi‐incompressible materials accurately and an implicit quasi‐static solver was used. A 5‐mm radius spherical indenter which simulates the instrumented palpation system was modeled as a discrete rigid solid, meshed with 3 node triangular and 4 node bilinear quadrilateral facets as shown in Figure 3. The connection of the indenter to other parts of the instrumented palpation system was not modeled in this study, because it often only consists of a small diameter cable 41 whose influence on the recorded force feedback is negligible. Surface to surface contact considering finite strains was used to simulate the interaction between the indenter and the rectal wall. The palpation was simulated under displacement control with a maximum allowable displacement of 5 mm.

Two modeling scenarios will be considered: completely healthy and completely cancerous prostate. This will allow the determination of the upper and lower limits of the instrumented digital rectal examination force feedback diagrams as well as investigating how the resolution (the distance between the force feedback curves for the cancerous and healthy scenarios) varies for different patients and IBPs.

## 3 Results and Discussion

In this section, the effects that subject specific features (e.g. geometry and structure) have on the outcome of instrumented digital rectal examination are explored. For each of the three cases, cancerous and healthy prostates are modeled to assess the sensitivity of the instrumented digital rectal examination to patient‐specific factors. Finally, the effects of different intrabladder pressures on diagnostic sensitivity are analyzed. First, the influence of anatomical (in pelvic cavity) and pathological (prostate) inter‐patient variation will be studied. Then the changes of intrabladder pressure will be considered. Finally, the presence of the pelvic bone in the model will be analyzed.

### 3.1 Patient‐specificity: anatomy and pathology

**Figure 4:**
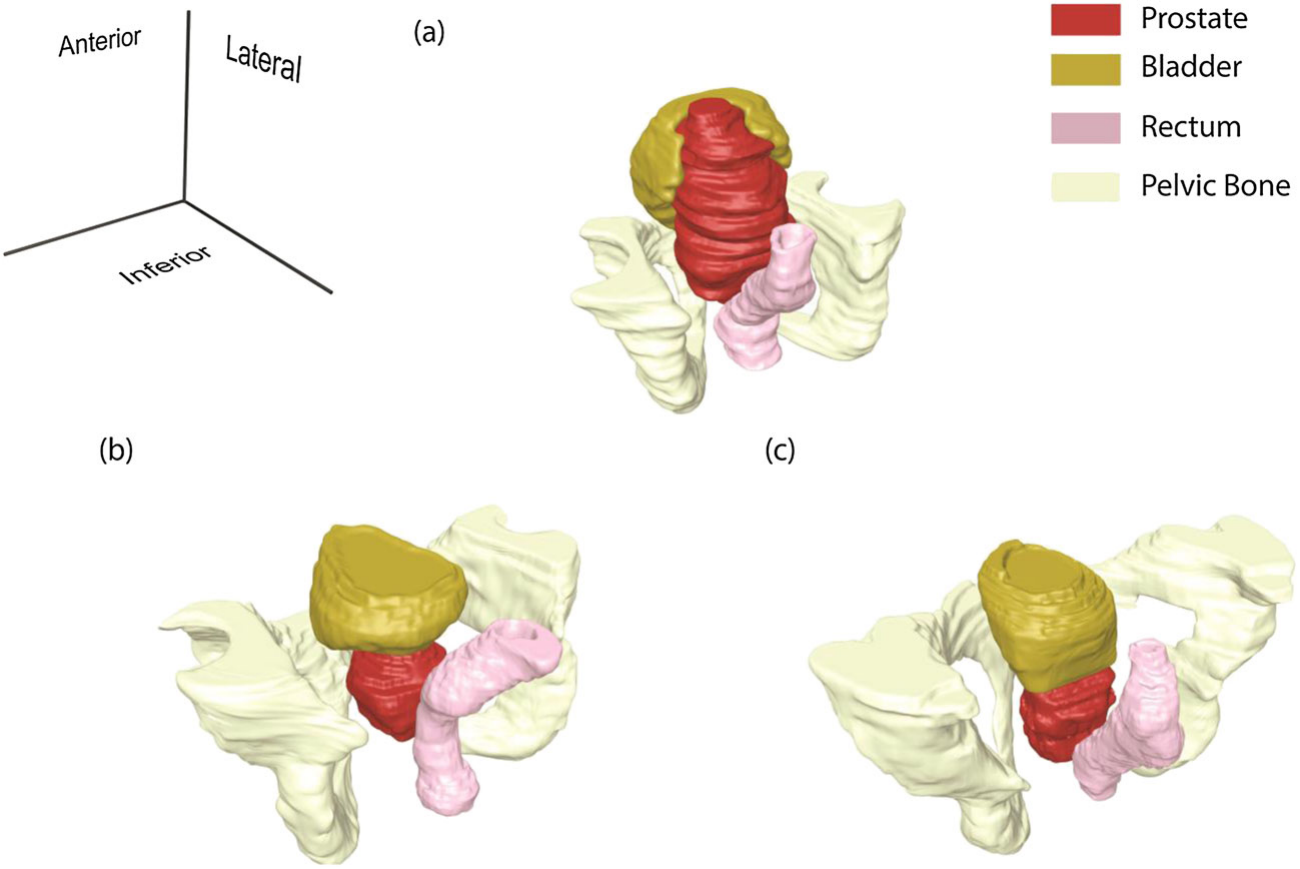
Reconstructed 3D models of three selected patients from MRI scans. (a) Patient 1; (b) Patient 2 and (c) Patient 3.

**Figure 5:**
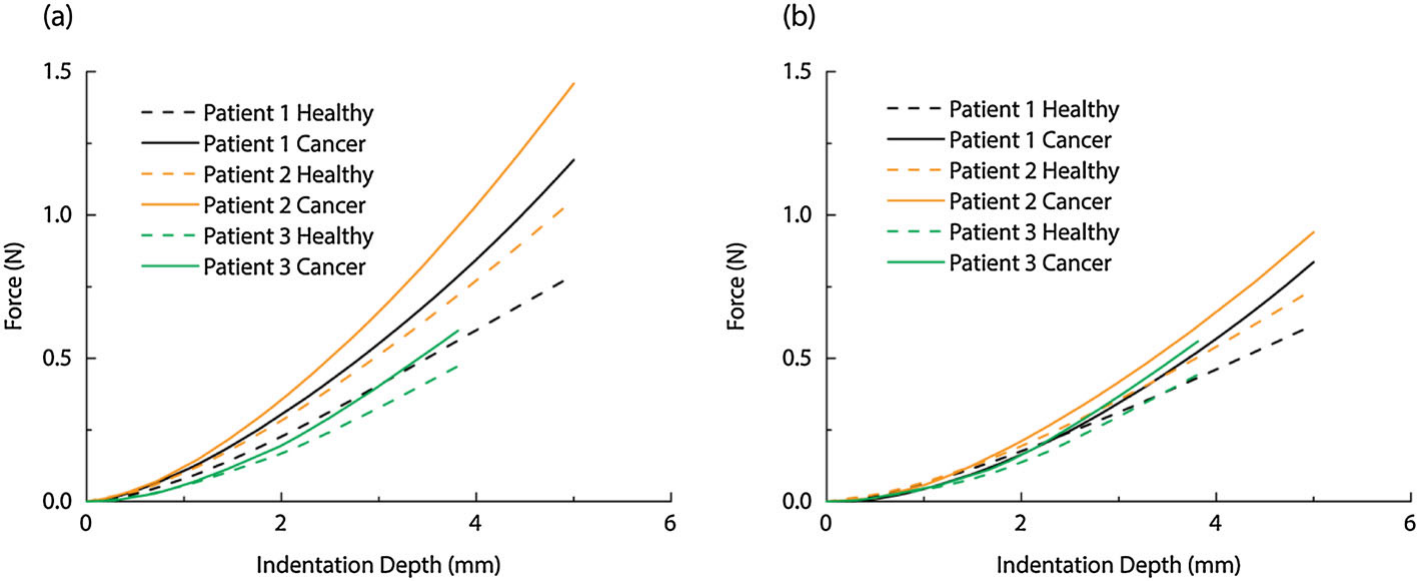
Changes in palpation force of three patients during IDRE subjected to (a) high intrabladder pressure and (b) low intrabladder pressure when the pelvic bone is considered.

In this section, in order to isolate the anatomical and pathological patient‐specificity, the presence of the pelvic bone is not considered. Figure 4 shows the reconstructed 3D models of the three patients from the MRI scans. It can be seen that the bladder of Patient 1 wraps around the enlarged prostate, Figure 4(a). Patient 2 has a different structure where the bladder sits above the prostate, as shown in Figure 4(b). Patient 3 is again different, where the bladder sits on top of the prostate with considerably less contact area, Figure 4(c). It should be noted here that the prostate of Patient 3 contains small holes (which are assumed to be filled with fascia) because of the brachytherapy treatment, which would potentially reduce its overall stiffness. Figure 5 shows the force feedback from the instrumented digital rectal examination with high and low intrabladder pressure, respectively, for all patients with each of the two tissue conditions when the pelvic bone is modeled. The curves for the force feedback of the fully healthy and fully cancerous tissue act as the lower and upper bounds of possible outcome of an IDRE test. The result of any test on a prostate with a cancerous nodule inside would lie within these two bounds so it represents the ‘diagnostic window’ against which resolution of percentage cancer could be assessed. The results for Patients 1 and 3 are comparable when the indentation depth is small, even though the overall stiffness of the prostate of Patient 3 is reduced because of the brachytherapy. This is potentially caused by the indentation being performed at the end part of the rectum, where only the inferior part of the prostate can be reached (even in DRE superior parts of the rectum are hardly reachable by the finger unless anesthesia is used). For larger depths of indentation the palpation force in Patient 2 becomes higher because a thicker region of the prostate is palpated. It can be seen that, although the reaction forces in the cancerous scenario are higher than in the healthy case, the relative difference between the cancer and healthy results remains approximately constant between different patients. When high intrabladder pressure is used, the differences between patients are intensified as shown in Figure 5(b). It is important to note that the diagnostic distinguishability of cancerous nodules during IDRE can be estimated by the vertical gap of force data between healthy and cancerous cases at certain indentation depth. It is clearly shown that the diagnostics would benefit from deeper palpation and also the presence of high intrabladder pressure.

It might be noted here that, at the beginning of the palpation modeling, the indenter is not in contact with the rectum. This can have the result that, at the very early stages of the indentation, no contact is registered, especially in the case of low intrabladder pressure. This is why the final indentation depth from the rectum is less than the 5 mm in the case of patient 3.

### 3.2 Variation of intrabladder pressure (IBP)

**Figure 6:**
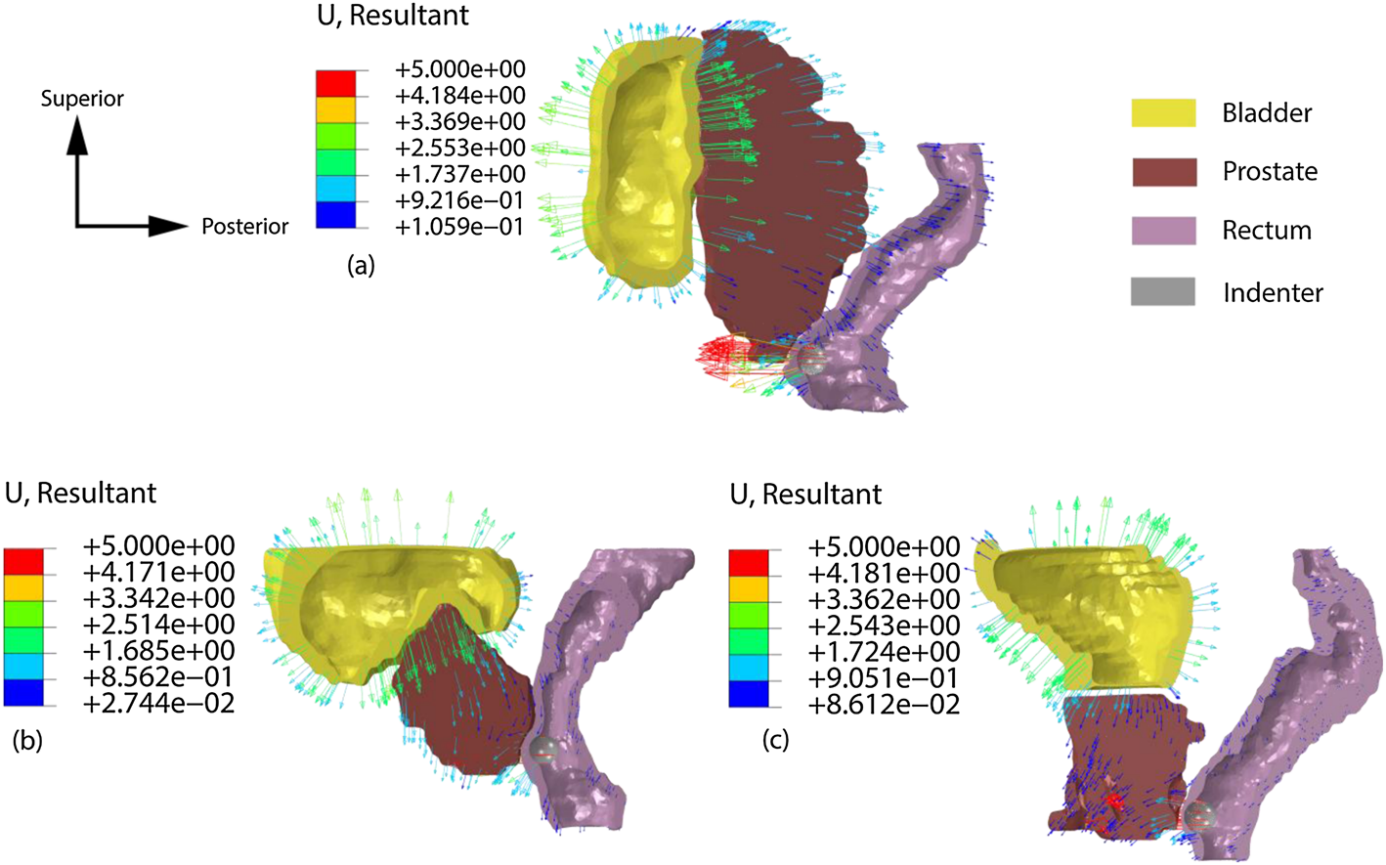
Displacement fields of three patients subjected to IDRE when LIBP is present. (a) Patient 1; (b) Patient 2 and (c) Patient 3. In these cases, the pelvic bone is not modeled, and the prostate is assumed to be healthy.

In this section the role of IBP will be investigated. The displacement fields for the three patients in IDRE tests when the intrabladder pressure is high are shown in Figure 6, in which it is possible to explore how a change in IBP influences the interaction between organs thus affecting the outcome of IDRE. Patient 1 has an enlarged prostate with the bladder surrounding it. This pushes the prostate towards the rectal wall which improves the probing. A similar phenomenon is seen in Patient 2, whose bladder surrounds the prostate which also results in a displacement towards the rectal wall when high intrabladder pressure is present. Patient 3 has a considerably different geometry where the bladder sits on top of the prostate without enveloping it. This causes a displacement of the prostate towards the anterior direction in the high intrabladder pressure condition. In all cases, the presence of IBP moves the prostate towards the inferior direction leading to an increased palpation area. For patients suffering from pollakiuria because of the enlargement of the prostate, performing the examination with higher IBP would be beneficial for the examination. More importantly, performing IDRE multiple times over a period of time with different IBP levels may help determine whether the bladder is being compressed by the prostate because of benign prostatic hyperplasia or a malignant neoplastic process.

**Figure 7:**
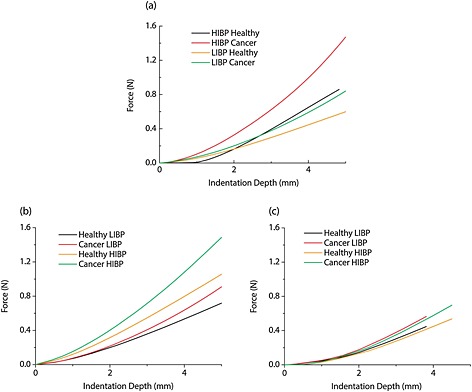
Reaction forces of Patients 1 (a), 2 (b) and 3 (c) under LIBP and HIBP conditions without the presence of the pelvic bone.

Figure 7 shows the force feedback for the three patient models subjected to IDRE, comparing the healthy and cancerous states under either low or high IBP conditions. As might be expected, the palpation force increases generally when high intrabladder pressure is present. More significantly, the distance between force data in the cancer and healthy cases also increases when IBP becomes higher, which could greatly improve the diagnostic distinguishability of the procedure. This is because of the structure of the pelvic cavity and interactions between organs which push the prostate against the rectal wall when the bladder is inflated. In contrast, the Patient 3 model exhibits a different behavior, in which the reaction forces are lower under the high intrabladder pressure as shown in Figure 7(b). Furthermore the resolution (i.e. the difference in force between the healthy and cancerous states) shows a weak dependency on the IBP.

### 3.3 Effects of pelvic bone

The male pelvic bone is subjected, among others, to loads caused by intrabladder pressure and, during DRE, by the palpation. The aim of this section is to understand the effects of presence of the pelvic bone in the prediction of the proposed patient‐specific model and determine if there are circumstances under which it needs to be modeled to obtain sufficiently accurate results. In this way, it will be possible to reduce unnecessary numerical problems associated with the inclusion of a stiff material in a soft matrix as well as reducing the uncertainty in the model.

**Figure 8:**
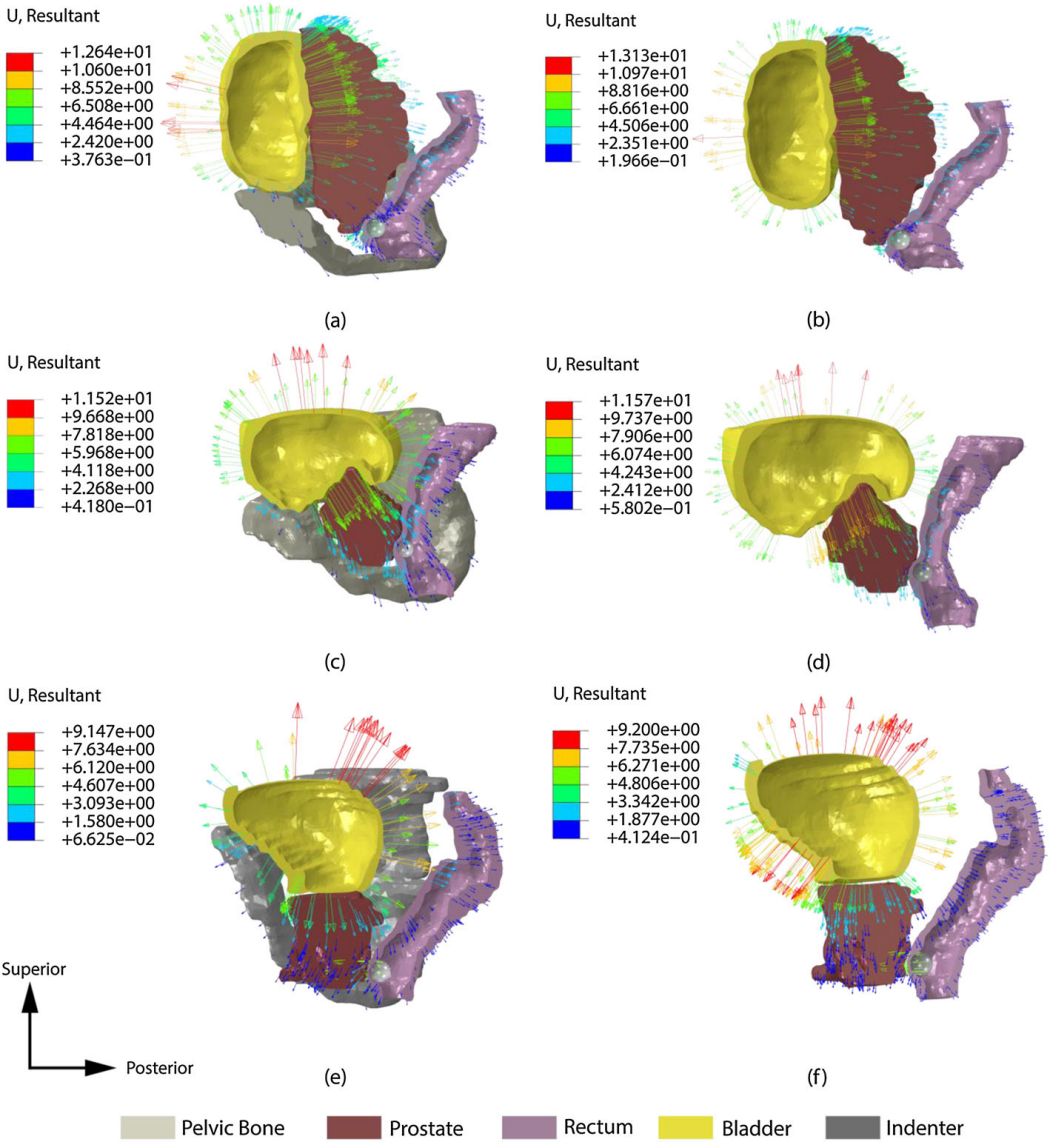
Displacement fields of Patients 1, 2 and 3, comparing the influence of the pelvic bone in IDRE under HIBP condition. The prostate is assumed to be completely healthy. (a) and (b): Patient 1 with and without pelvic bone, respectively; (c) and (d): Patient 2 with and without pelvic bone, respectively; (e) and (f): Patient 3 with and without pelvic bone, respectively.

Figures 8(a) and (b) show the displacement map of Patient 1 when the pelvic bone is present and absent, respectively. In this particular case the bladder sits on top of the pelvic bone and so the anteroposterior axis is not highly constrained by the bone except in the inferior part. This is critical in this particular case, because the prostate is moved along the inferoposterior direction, whereas, without the pelvic bone, the prostate is pushed directly towards the rectum. The effect of modeling the pelvic bone in Patient 2 is more significant in the inferior zone of the bladder that expands more freely when the bone is not considered. However, the deformation in the anteroposterior axis is not greatly influenced, because of the bladder being located over the bone so that its expansion is not highly constrained. The bladder, which partially envelops the prostate, forces it to move towards the rectum, with little constraint from the bone, because the bladder and prostate interact along the craniocaudal and anteroposterior axes. Patient 3 has a different structure again, where the bladder anterior side is surrounded by the pelvic bone, which hinders its expansion and causes a larger displacement of the prostate towards the bottom of the pelvic bone, as shown in Figures 8(c) and (d). For this patient, it is important to note that weak bladder‐prostate interaction in the anteroposterior axis causes only small displacements of the prostate along this axis.

**Figure 9:**
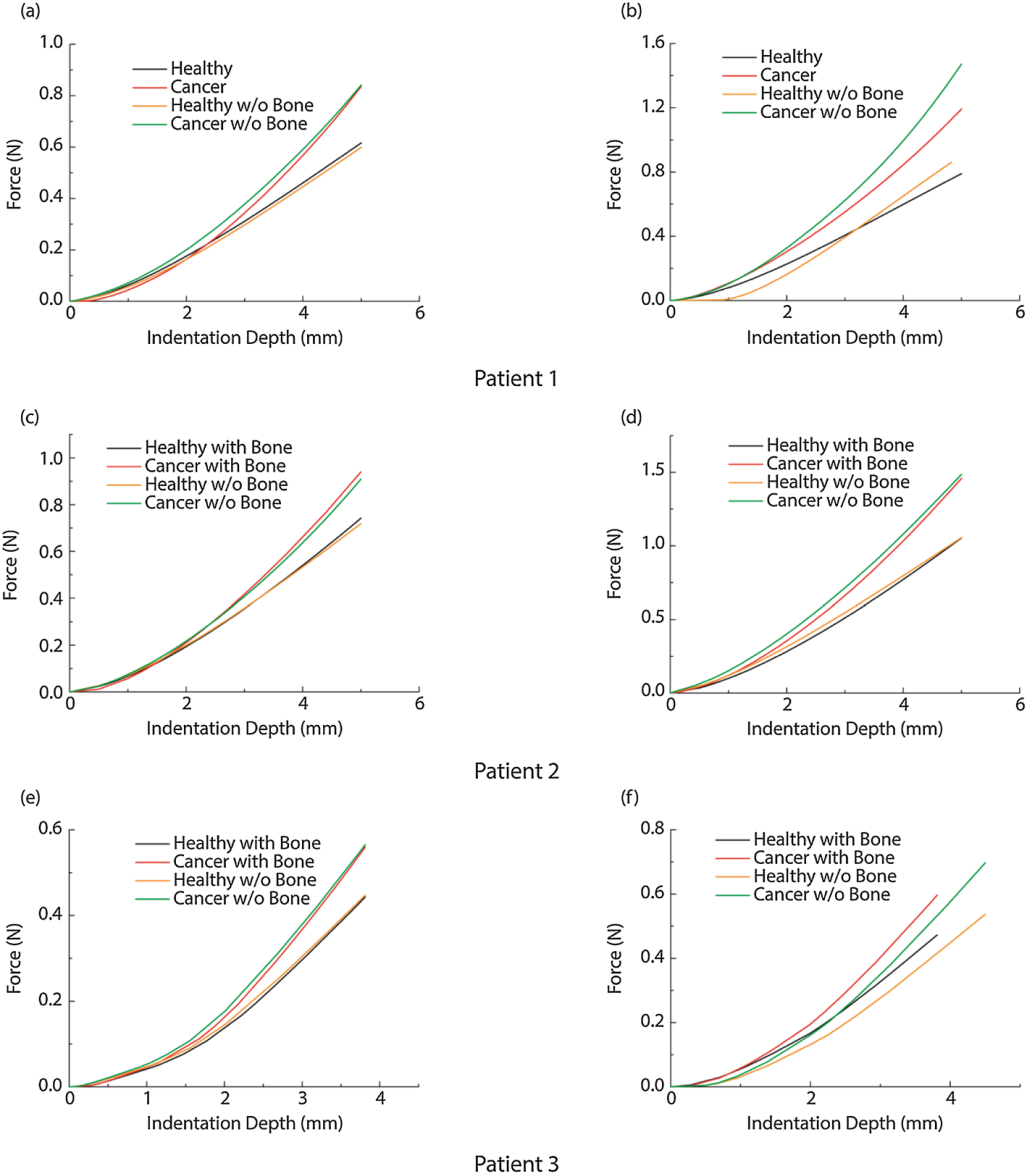
Comparison of the palpation forces of all three patients under low intrabladder pressure (left column) and high intrabladder pressure (right column) conditions. (a) and (b): Patient 1; (c) and (d): Patient 2 and (e) and (f): Patient 3. The results show a significant difference when the pelvic bone is not present.

Figures 9(a) and (b) show the palpation force data for Patient 1 under high and low IBP conditions. The effect of modeling the pelvic bone is small, especially when the IBP is low but becomes more significant when high intrabladder pressure is present. For the Patient 2 model there is little difference when the pelvic bone is not modeled as shown in Figures 7(c) and (d). Therefore, for this patient, in whom the bladder wraps around and on top of the prostate forcing it to move towards the area where palpation is performed, the presence of the pelvic bone would be expected to have little influence in the outcome of instrumented DRE making it unnecessary to take it into account in the patient‐specific model. Finally, for Patient 3 the differences are very small under the low intrabladder condition. On the other hand, for high intrabladder pressure, the force feedback of the instrumented DRE significantly increases because of the aforementioned effect of the pelvic bone hindering the expansion of the bladder along the anteroposterior axis.

## 4 Conclusion

This paper aimed to study the effects of patient specific features, e.g. organ geometry and intrabladder pressure, in the outcome of instrumented digital rectal examination. The following key conclusions are highlighted:

1. Higher intrabladder pressure, which could be achieved by asking the patient to retain urine prior to the examination, would improve the sensitivity of the procedure.
2. The pelvic bone significantly affects the force feedback of the digital rectal examination and therefore needs to be modeled, especially when high intrabladder pressure is present.
3. Subject specific structural features (caused by previous treatments) could influence the diagnostic outcome, and therefore need to be incorporated in the framework of patient‐specific modeling as well as quantitative diagnostic procedures.
4. The relative position of the bladder and prostate significantly affects the force feedback and, consequently, the sensitivity of the procedure, in particular when high intrabladder pressure is present.

The proposed framework has certain limitations as it stands. Some organs, like the seminal vesicles, urethra and neuromuscular bundle have not been considered because the resolution of the MRI images obtained from standard clinical protocol is insufficient to obtain an accurate representation of them. Complex interactions such as slip between fat and organs and muscle–bone connections are not taken into account because of the impracticality of doing so in clinical practice. Furthermore, it was assumed that the tissues are isotropic and the intra‐patient variations of their properties are not taken into account in this study. It is hoped that, as a part of future work, the clinical validation of this framework will be conducted once all the data from the instrumented digital rectal examination has been collected. Ultimately, it is expected that such an approach will allow a reduction in the number of expensive and invasive biopsies.
